# Integrative Multi-Omics Analysis Identifies Tissue, Cellular and Splicing Programs Associated with Exercise-Mediated Improvement in Type 2 Diabetes

**DOI:** 10.3390/cells15110979

**Published:** 2026-05-26

**Authors:** Jingzhe Xiao, Yuwei Ding, Songbo Li, Yi Yan, Ziyue Yu, Pengyu Fu, Chunyan Xu, Lijing Gong

**Affiliations:** 1 China Ice Sport College, Beijing Sport University, Beijing 100084, China; xiaojingzhe1120@bsu.edu.cn; 2 Key Laboratory of Physical Fitness and Exercise, Ministry of Education, Beijing Sport University, Beijing 100084, China; 3 State Key Laboratory of Bioactive Substance and Function of Natural Medicines, Institute of Materia Medica, Chinese Academy of Medical Sciences and Peking Union Medical College, Beijing 100050, China; dingyuwei@imm.ac.cn; 4 School of Sport Science, Beijing Sport University, Beijing 100084, China; bsuedison@163.com (S.L.); yanyi@bsu.edu.cn (Y.Y.); 5 School of Sports Medicine and Rehabilitation, Beijing Sport University, Beijing 100084, China; 2022015241@bsu.edu.cn; 6 Department of Physical Education, Northwestern Polytechnical University, Xi’an 710072, China; fupy@nwpu.edu.cn; 7 Beijing Sports Nutrition Engineering Research Center, Beijing 100084, China; 8 Key Laboratory for Performance Training & Recovery of General Administration of Sport, Beijing 100084, China

**Keywords:** diabetes, exercise, physical activity, genome-wide association study, single cell, single-cell RNA sequencing

## Abstract

**Highlights:**

**What are the main findings?**

- Cross-tissue and single-cell analyses highlighted skeletal muscle and adipose tissue as major tissues involved in exercise–T2D crosstalk.
- Candidate cell populations, pathways, and splicing changes associated with exercise-responsive metabolic remodeling were identified and supported by mouse data.

**What are the implications of the main findings?**

- The results refine current understanding of how exercise-related regulatory programs may be linked to T2D biology across multiple levels.
- The prioritized tissues, cell types, and pathways provide a basis for future functional validation and intervention studies.

**Abstract:**

Physical inactivity contributes to type 2 diabetes (T2D), but the molecular links between exercise and metabolic improvement remain incompletely understood. We meta-analyzed genome-wide association studies of vigorous physical activity and T2D (combined *n* ≈ 1.95 million) and integrated eQTL/sQTL maps with single-cell and spatial transcriptomic datasets to connect genetic risk with tissues, cell types, and regulatory programs. Tissue and cell-type enrichment, colocalization, and network analyses were performed. Computational findings were further examined in male 10-week-old C57BL/6J mice with high-fat diet-induced diabetes. After 1 week of acclimatization, mice were randomly assigned to normal chow, high-fat diet, or high-fat diet plus exercise groups (n = 6 per group; high-fat diet with 60% of total energy from fat). The exercise intervention consisted of treadmill running (10 m/min for 50 min per day, 5 days per week, total 16 weeks), followed by metabolic phenotyping, skeletal muscle histology, bulk RNA sequencing, alternative splicing analysis, and RT-qPCR of *Mau2* isoforms. Exercise- and T2D-associated variants showed joint enrichment in skeletal muscle and adipose eQTL/sQTL signals. Integrated single-cell analyses prioritized fibro-adipogenic progenitors and endothelial cells, and identified an extracellular matrix- and collagen-related module in fibro-adipogenic progenitors associated with both exercise and T2D. *Mau2* emerged as a shared candidate gene with tissue-specific splicing signals. In diabetic mice, exercise improved glucose homeostasis and muscle fiber structure, and reduced *Mau2* intron retention in skeletal muscle without changing total *Mau2* expression. These findings support a multiscale framework linking exercise-responsive regulation to T2D-related tissue remodeling and splicing plasticity.

## 1. Introduction

Physical activity is fundamental for human energy balance, social interaction, and organ function. From a metabolic perspective, regular exercise maintains glucose–lipid homeostasis by improving insulin sensitivity, mitochondrial biogenesis, and oxidative phosphorylation [1,2,3], and it remodels endocrine and cytokine networks across multiple organs [4]. At the population level, physical activity is a cornerstone in the prevention of chronic non-communicable diseases [5]. However, in contemporary societies characterized by convenience and sedentary behavior, type 2 diabetes (T2D)—defined by insulin resistance, β-cell dysfunction, and chronic low-grade inflammation—has become one of the most prevalent and burdensome metabolic diseases worldwide [6,7,8]. Its impact extends far beyond hyperglycemia: skeletal muscle, liver, visceral and subcutaneous adipose tissue, pancreatic islets, the vasculature, and the central nervous system all undergo coordinated structural and functional injury. These changes include impaired muscular glucose uptake and fatty acid oxidation, exacerbated hepatic lipotoxicity and ectopic fat deposition, increased immune cell infiltration and dysregulated lipolysis in adipose tissue, and islet stress with failure of compensatory insulin secretion, ultimately driving and amplifying the risk of T2D-related complications [9,10,11].

Despite continuous advances in pharmacotherapy, exercise remains a cornerstone of T2D prevention and management, improving glycemic control, cardiorespiratory fitness, systemic inflammation, and body composition [12,13]. However, metabolic responses to exercise vary substantially among individuals, and the molecular and cellular basis of this heterogeneity remains incompletely defined. Because T2D involves coordinated dysfunction across skeletal muscle, liver, adipose tissue, pancreatic islets, immune signaling, and mitochondrial regulation [14,15,16], single-layer analyses are insufficient to capture the biological interface between exercise and T2D. This highlights the need for integrative multi-omics approaches to identify tissues, cell types, and regulatory programs that may contribute to exercise-related metabolic improvement [17].

Multiple genome-wide association studies (GWAS) of physical activity and metabolic traits have identified overlapping loci, including CADM2, a robust PA-associated gene that has also been linked to obesity and related metabolic traits. These findings suggest that PA genetics may involve both behavioral regulation and metabolic biology, supporting the need to further dissect how PA-associated signals connect with T2D-related tissues and regulatory programs [17,18,19]. However, it remains unclear how these shared and distinct genetic effects are mediated through expression quantitative trait loci (eQTLs) and splicing quantitative trait loci (sQTLs) across human tissues, and how they converge on specific cell types, gene networks, and signaling pathways to shape clinical phenotypes. Single-layer study designs cannot resolve these multi-scale mechanisms, underscoring the need to integrate genetic data with multi-omics functional readouts. To systematically dissect the relationship between exercise and T2D, we leveraged large-scale GWAS summary statistics and integrated them with multi-layer datasets, including single-cell RNA sequencing (scRNA-seq), spatial transcriptomics, and single-cell chromatin accessibility profiles. Using state-of-the-art cross-omics methods, we interrogated shared and trait-specific biological features of exercise and T2D across system-level, cellular, and molecular dimensions.

In this study, we integrated large-scale GWAS summary statistics for vigorous physical activity and T2D with GTEx eQTL/sQTL maps, single-cell RNA-seq, spatial transcriptomics, and cell type prioritization analyses. We aimed to identify tissues, cell populations, gene networks, and splicing events associated with the biological interface between exercise and T2D. Selected computational findings were further examined in a mouse exercise model. This framework was designed to generate biologically interpretable and experimentally supported hypotheses, rather than to establish definitive causality.

## 2. Materials and Methods

**Figure 1:**
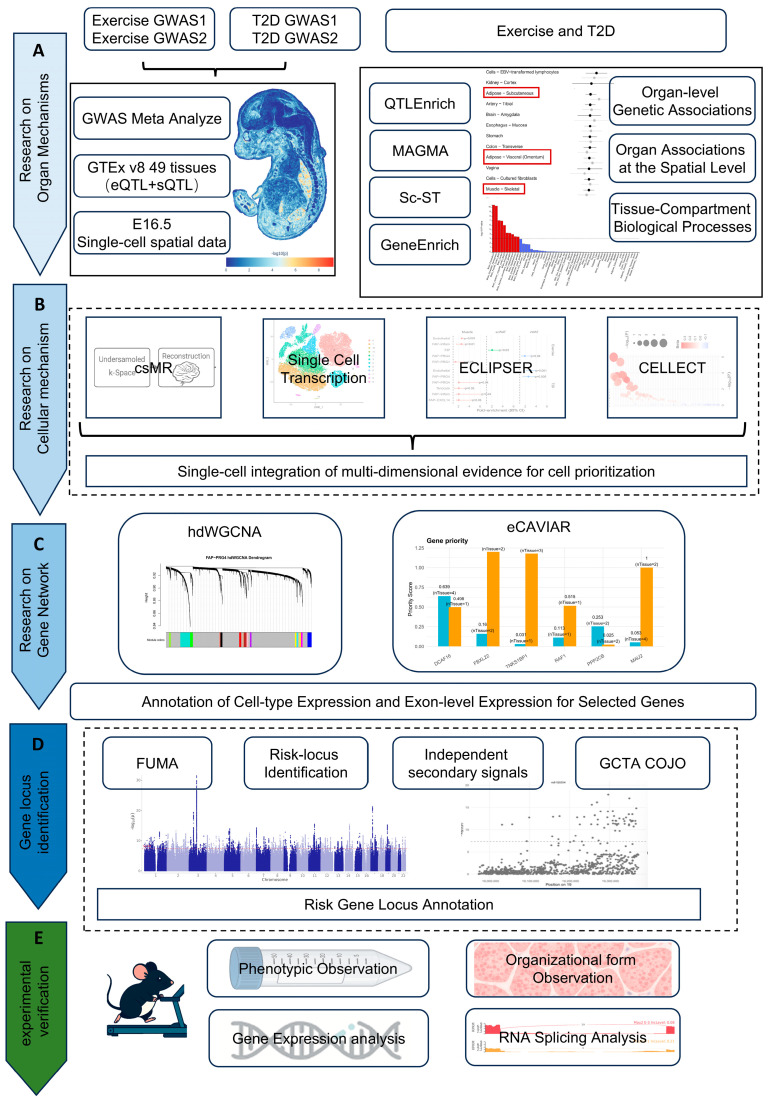
Integrative multi-level analytical workflow to investigate how exercise ameliorates T2D. (**A**) Organ/tissue level: Starting from GWAS of exercise-related traits and T2D, we combined GTEx v8 eQTL/sQTL data with E16.5 single-cell spatial transcriptomic data and used QTLEnrich, MAGMA, sc-ST, and GeneEnrich to characterize tissue- and compartment-specific genetic enrichment and related biological processes. (**B**) Cellular level: We integrated multiple single-cell lines of evidence (cell type-specific Mendelian randomization [csMR], single-cell transcriptomics, ECLIPSER, and CELLECT) to prioritize candidate cell types. (**C**) Gene-network level: Within prioritized cell populations, we applied hdWGCNA to construct weighted gene co-expression networks and used eCAVIAR for cross-study colocalization to nominate candidate genes. (**D**) Locus level: We performed locus and gene annotation using FUMA, conditional/independent signal analysis, and GCTA-COJO. (**E**) Experimental validation: We validated computational findings in mouse models using phenotypic assessments, histology, gene expression analysis, and RNA alternative splicing assays.

All data included in this study comply with all relevant ethical standards. Detailed information is provided in Supplementary Table S1. This study followed a hierarchical five-step integrative workflow, with each layer serving a distinct purpose: tissue-level discovery, spatial and functional annotation, cell-type prioritization, gene/locus refinement, and experimental support. At the organ level, we performed meta-analyses of two exercise GWAS and two T2D GWAS and integrated the summary statistics with GTEx v8 eQTL and sQTL data from 49 tissues and E16.5 single-cell spatial transcriptomic data. Using QTLEnrich, Multi-marker Analysis of GenoMic Annotation (MAGMA, v1.10), single-cell spatial transcriptomics (Sc-ST) and GeneEnrich (web-based platform; accessed on 25 December 2025), we mapped genetic associations to organs, spatial compartments and tissue-compartment-specific biological processes (Figure 1A). At the cellular level, we combined single-cell transcriptomic data with csMR (implemented in Fast2TWAS, version 0.0.26.5), ECLIPSER (implemented in Fast2TWAS, version 0.0.26.5) and CELLECT (implemented in Fast2TWAS, version 0.0.26.5) to integrate multi-dimensional evidence and prioritize cell types relevant to exercise and T2D (Figure 1B). At the gene-network level, hdWGCNA (R package version 0.4.7) and eCAVIAR (Linux version 2.0.0) were used to construct co-expression networks, identify key modules and annotate cell-type-specific and exon-level expression patterns of selected genes (Figure 1C). For risk locus refinement, FUMA (web-based platform; accessed on 25 December 2025) and GCTA-COJO (web-based platform; accessed on 25 December 2025) were applied to identify independent secondary signals and functionally annotate risk gene loci (Figure 1D). Finally, in mouse aerobic exercise models we performed phenotypic assessments, histological examinations, gene expression profiling and RNA splicing analyses to experimentally support main results (Figure 1E). Because these analytical layers used different input units, statistical models and null hypotheses, they were not treated as a single unified statistical test. Instead, method-specific multiple-testing corrections were applied within each layer, and cross-layer convergence was interpreted as prioritization evidence.

### 2.1. Sources of Genome-Wide Summary Statistics

GWAS summary statistics for exercise-related traits were obtained from the IEU OpenGWAS resource, including (i) Strenuous sports or other exercises (GCST006100; n = 350,492; 124,842 cases and 225,650 controls) and (ii) Other exercises (e.g., swimming, cycling, keep fit, bowling) (ukb-b-8764; n = 460,376; 222,470 cases and 237,906 controls). We meta-analyzed these two datasets to represent vigorous/strenuous physical activity (total n = 810,868).

For T2D, we combined (i) Type 2 diabetes (ebi-a-GCST006867; n = 655,666; 61,714 cases and 593,952 controls) and (ii) FinnGen Release 12 T2D (FINNGEN_R12_T2D; n = 486,367; 82,878 cases and 403,489 controls). These datasets were meta-analyzed for downstream analyses (total n = 1,142,033).

### 2.2. Single-Cell Transcriptomic Data

We used publicly available scRNA-seq data from GSE183288, which profiled subcutaneous adipose tissue, visceral adipose tissue, and skeletal muscle from mice with high-fat diet-induced T2D with or without exercise intervention (*n* = 4 mice per group for each tissue) using the 10x Genomics Chromium platform [20]. The original study generated gene–cell UMI count matrices with Cell Ranger (v3.1.0) after Illumina sequencing. We downloaded the processed count matrices and metadata from GEO and performed quality control, normalization, integration, clustering, and cell-type annotation using Seurat (v4.x) to interrogate cellular features and transcriptional programs associated with exercise and T2D.

### 2.3. Quality Control

A series of quality control (QC) procedures was applied to both GWAS and scRNA-seq data to ensure the accuracy and reliability of downstream analyses.

For the GWAS data, variants were first filtered based on minor allele frequency (MAF). SNPs with MAF < 0.01 were removed to exclude low-frequency variants that could increase uncertainty and inflate false-positive findings. To avoid spurious associations driven by extensive linkage disequilibrium (LD) in the major histocompatibility complex (MHC) region on chromosome 6, SNPs within the MHC region were excluded. All GWAS summary statistics were then harmonized to a unified reference genome build and standardized file format prior to analysis.

For the scRNA-seq data, a systematic QC pipeline was implemented using Seurat. For each cell, the percentage of mitochondrial genes (prefixed “^MT-“) and hemoglobin genes (including HBA1, HBA2, HBB and related loci) was calculated to assess cell quality. High-quality cells were retained using stringent thresholds: ≥1000 unique molecular identifiers (UMIs), 200–5000 detected genes, mitochondrial gene content ≤15%, and hemoglobin gene content ≤3%. QC metrics before and after filtering were visualized using violin plots. Dimensionality reduction was performed using principal component analysis (PCA) followed by t-distributed stochastic neighbor embedding (t-SNE), and the contribution and stability of principal components were evaluated using heatmaps and elbow plots. Batch effects across samples were corrected with the Harmony algorithm (theta = 2, lambda = 1). Software versions, key parameter settings and key analysis scripts are available through the GitHub repository listed in the Data Availability Statement.

### 2.4. Genome-Wide Meta-Analysis

To increase statistical power, GWAS meta-analyses were performed by combining summary statistics across cohorts for the exercise and T2D GWAS. This integration was intended to enhance the detection of genetic variants with modest effect sizes. Between-study heterogeneity was quantified using the I^2^ statistic, which reflects the proportion of total variance attributable to heterogeneity rather than sampling error. When I^2^ exceeded 50%, indicating moderate to substantial heterogeneity, random-effects models were considered in the meta-analysis.

### 2.5. Tissue-Specific eQTL/sQTL Enrichment for Exercise and T2D Using QTLEnrich

To evaluate tissue-specific enrichment of genetic associations for exercise and T2D, expression quantitative trait loci (eQTLs) and splicing quantitative trait loci (sQTLs) from 49 tissues in GTEx v8 were used. QTLEnrich is a rank- and permutation-based method that tests whether phenotype-associated variants are enriched among tissue-specific eQTLs and sQTLs and quantifies the statistical significance of this enrichment. The method explicitly accounts for three potential confounders: MAF, distance to the transcription start site (TSS) of the target gene, and local LD.

Adjusted fold enrichment (Adjusted_Fold_Enrichment) and the enrichment *p* value (Enrichment_P_value) reported by QTLEnrich were used to assess the magnitude and significance of tissue-specific eQTL/sQTL enrichment. Multiple testing and enrichment significance were evaluated according to the permutation-based framework implemented in QTLEnrich. To further evaluate the significance and robustness of QTLEnrich results, association *p* values between each SNP and the phenotype were obtained and transformed to −log_10_(P). Quantile–quantile (Q–Q) plots were then generated by plotting observed −log_10_(P) values against expected −log_10_(P) values under the null hypothesis of no association (uniform distribution), providing a visual assessment of enrichment.

To complement QTLEnrich and broaden the characterization of genomic features related to exercise and T2D, additional enrichment analyses were performed using MAGMA. GWAS summary statistics for exercise and T2D were reformatted according to MAGMA input requirements, and gene-level association tests were conducted. Because QTLEnrich and MAGMA evaluate different statistical units and hypotheses, their *p* values were not pooled into a single cross-method global FDR. Instead, tissue-level signals were interpreted according to method-specific thresholds and cross-method consistency. Tissue-specific enrichment signals with *p* < 0.01 were considered supportive evidence for downstream tissue prioritization.

### 2.6. Tissue-Specific Spatial Enrichment of Exercise- and T2D-Associated Signals in Single-Cell Spatial Transcriptomics

To investigate the spatial context of exercise- and T2D-associated signals, sc-ST data were integrated with GWAS summary statistics to map the spatial distribution of trait-associated cells at single-cell resolution. The genetically informed spatial mapping algorithm gsMap was applied to perform cross-species integration of mouse embryonic/brain tissues, macaque cerebral cortex, and human GWAS data, thereby identifying disease-relevant cellular populations and their spatial organization.

Conceptually, gsMap uses GWAS-derived trait-associated genes and projects their expression patterns onto spatially resolved cells, quantifying associations between specific anatomical regions and complex traits at the cellular level. Using a spatial transcriptomic atlas of E16.5 mouse embryos covering 25 organs, spatial enrichment maps for exercise and T2D were generated, alongside corresponding spatial gene expression maps. These analyses were used to construct a single-cell-resolution spatial map of T2D pathogenesis.

### 2.7. GeneEnrich Analysis of Biological Processes in Adipose Tissue and Skeletal Muscle Related to Exercise and T2D

Gene set enrichment analysis was applied to systematically interrogate genetic variants and expression profiles associated with exercise and T2D. GeneEnrich, which combines hypergeometric testing with permutation-based assessment, was used to evaluate whether candidate gene sets were enriched in tissue-related biological process or phenotype gene sets. To reduce tissue-specific bias, empirical *p* values were derived from permutation tests. To minimize confounding commonly encountered in genetic analyses, genes located within the MHC region were excluded.

Analyses focused on three tissue compartments: (1) subcutaneous adipose tissue regions associated with exercise and T2D; (2) visceral adipose tissue regions associated with exercise and T2D; and (3) skeletal muscle regions associated with exercise and T2D. Functional gene sets were obtained from Gene Ontology (GO), Reactome, Kyoto Encyclopedia of Genes and Genomes (KEGG), the Molecular Signatures Database (MSigDB), and Mouse Genome Informatics (MGI). Within each database, empirical *p* < 0.05 was considered nominally significant.

### 2.8. Single-Cell Cell Type-Specific Mendelian Randomization (csMR) Analysis

To further dissect cell type-specific effects, single-cell eQTL data were integrated with a Mendelian randomization (MR) framework to evaluate how genetic variants acting in specific cell types may causally influence disease risk. The cell type-specific MR (csMR) framework was used to infer putative causal cell types for exercise-related traits and T2D. Specifically, the single-cell eQTL dataset reported by Hao et al. was used to perform csMR analyses across eight major cell types: astrocytes, endothelial cells, excitatory neurons, inhibitory neurons, microglia, oligodendrocytes, oligodendrocyte precursor cells, and pericytes [21].

### 2.9. Identification of Exercise- and T2D-Related Cellular Signals: Annotation of the Single-Cell Transcriptomic Atlas

In-depth analyses were performed on preprocessed scRNA-seq data. Using Harmony-corrected embeddings, multidimensional visualization was conducted, including PC heatmaps, elbow plots, and two-dimensional projections based on t-SNE and Harmony. Cell-cycle distribution was assessed by calculating S-phase and G2/M-phase scores to quantify cell-cycle states. A cell–cell neighborhood graph was constructed using the Harmony embeddings, and a multi-resolution clustering strategy was applied, testing resolutions from 0.01 to 3.0 (13 resolutions in total). Based on the cluster tree, a resolution of 0.8 was selected as optimal, yielding stable cell subpopulations. To characterize the molecular features of each cluster, the Seurat function FindAllMarkers was used with a minimum expression fraction of 25% (min.pct = 0.25) and a log fold-change threshold of 0.25 (logfc.threshold = 0.25) to identify cluster-specific marker genes. These marker genes were visualized in heatmaps and used for cell type annotation.

### 2.10. Identification of Exercise- and T2D-Related Cellular Signals Using ECLIPSER

ECLIPSER was used to test whether GWAS loci for exercise-related traits and T2D are preferentially linked to genes with cell type-specific expression. For each trait, GWAS lead variants were clumped into loci and expanded with LD proxies (r^2^ > 0.8) to capture correlated variants. ECLIPSER quantifies cell type-specific enrichment by contrasting the proportion of trait loci mapped to cell type-specific genes against a background set of GWAS loci, using a Bayesian extension of Fisher’s exact test. The output includes a fold-enrichment estimate with a 95% confidence interval and an enrichment *p* value for each trait–tissue–cell type combination.

Cell type-specific genes were defined from within-tissue differential expression. Differential expression was computed using Wilcoxon rank-sum tests with a minimum of three cells per group, a minimum detection rate of 10% per gene, and |log_2_ fold change| > 0.5. Genes were considered cell type-specific if they met BH-adjusted *p* < 0.05 and the effect-size threshold. Loci were then scored for each cell type, and the cell type-specific locus set was defined using the 95th percentile of locus scores observed in the background GWAS locus set. Enrichment *p* values across tested cell types were adjusted using BH correction, and BH-adjusted *p* ≤ 0.05 was used to define significant enrichment.

### 2.11. Identification of Exercise- and T2D-Related Cellular Signals Using CELLECT

CELLECT was used to evaluate whether cell type-specific expression patterns explain GWAS heritability for exercise-related traits and T2D through two complementary models: stratified LD score regression (S-LDSC) and MAGMA. Cell type annotations were derived from public single-cell reference datasets (tabula_muris-test and mousebrain-test), and GWAS LD scores were computed using European-ancestry samples from the 1000 Genomes Project Phase 3 reference panel.

For S-LDSC, CELLECT converts cell type-specific gene expression into SNP-level annotations and fits a baseline model augmented with each cell type annotation to estimate the contribution of that annotation to trait heritability. The effect size (β) and corresponding *p* value were reported per cell type, and *p* < 0.05 was used to define nominally supportive enrichment. In parallel, MAGMA tested whether genes with higher expression in a given cell type show stronger gene-level association signals, reporting an effect estimate and *p* value for each cell type; *p* < 0.05 was used as the nominal significance threshold.

### 2.12. Methods for Integrated Single-Cell Evidence and Cell Type Prioritization

To prioritize candidate cell types associated with exercise- and T2D-related biology, four complementary lines of evidence were integrated: (i) atlas-based cell type annotation, (ii) cell type-specific MR (csMR) analysis, (iii) ECLIPSER, and (iv) CELLECT. Because these approaches differ in input data, statistical assumptions, and effect estimates, they were not combined using a formal meta-analytic weighting model. We used a transparent evidence-counting framework based on predefined evidence units. In the single-cell atlas layer, skeletal muscle and adipose tissue were treated as two separate tissue-level evidence units, because the same cell class identified across both tissues provided broader cross-tissue support than identification in only one tissue. Therefore, the single-cell atlas layer could contribute 0, 1, or 2 points. The total score for each cell type therefore reflected the number of positive evidence units supporting each cell type, rather than a formal statistical weight, probability estimate, causal effect size, or meta-analytic result. Higher scores indicate stronger cross-method and cross-tissue convergence and were used only for candidate cell-type prioritization. A score of 0 indicated that the corresponding evidence unit did not meet its predefined support criterion and was not interpreted as negative evidence or as a penalty in the final prioritization scheme [22].

### 2.13. Weighted Gene Co-Expression Network Analysis in Prioritized Cell Types to Identify Core Module Genes

The hdWGCNA framework was applied to perform weighted gene co-expression network analysis (WGCNA) in the intersecting cell types prioritized by the integrated single-cell evidence. Annotated single-cell Seurat objects were loaded and target cell types were specified for analysis. The WGCNA environment was initialized, and genes expressed in at least 5% of cells were selected as the candidate gene set.

To reduce the sparsity of single-cell data while preserving biological variability, metacells (k = 25) were constructed using the MetacellsByGroups function based on cell type and sample of origin, followed by normalization and standardization of metacell expression profiles. After dimensionality reduction by PCA, the Harmony algorithm was applied to correct batch effects related to sample origin.

To construct gene co-expression networks, the SetDatExpr function was used to extract the expression matrix for the target cell types, and the TestSoftPowers function was employed to evaluate candidate soft-thresholding powers. An appropriate soft-thresholding power was selected to build a signed topological overlap matrix (TOM). Co-expression modules were identified using a dynamic tree-cutting algorithm, and module eigengenes were calculated for each module. Relationships between modules and cell types were then assessed, within-module connectivity was evaluated, and modules were renamed to enhance biological interpretability.

### 2.14. Genomic Risk Locus Analysis for Exercise and T2D

FUMA (Functional Mapping and Annotation of GWAS) was used to identify and annotate genomic risk loci for exercise-related traits and T2D. Meta-analysis GWAS summary statistics, including SNP identifiers and LD reference information, were uploaded in the required format. FUMA performed initial quality control by removing SNPs with missing values or low data quality. A genome-wide significance threshold of *p* < 5 × 10^−8^ was then applied to define genomic risk loci associated with exercise and T2D.

### 2.15. Conditional Analysis of Genomic Risk Loci for Exercise and T2D

Conditional analyses were performed on high-confidence genomic risk loci for exercise-related traits and T2D to determine whether independent secondary signals colocalize within loci showing significant GWAS associations. For each locus, the lead variant was treated as a conditioning variable, and the COJO module of GCTA was used to conduct conditional association analyses with the GWAS summary statistics for exercise and T2D. To ensure that low-frequency lead variants were retained, variants with MAF < 0.0001 were removed before analysis.

### 2.16. eCAVIAR Analysis of Genomic Colocalization for Exercise and T2D

To identify high-confidence genes and regulatory mechanisms (eQTL/sQTL) potentially underlying shared risk loci for exercise-related traits and T2D, the Bayesian colocalization method eCAVIAR was applied. eCAVIAR evaluates whether overlapping GWAS and e/sQTL signals are likely to tag the same causal variant or haplotype, accounting for local LD and allelic heterogeneity. The method includes an internal fine-mapping procedure and can handle large GWAS summary statistics without requiring individual-level genotype data. In these analyses, at most two independent causal variants per locus were assumed.

As input to eCAVIAR, Z scores for each variant from the GWAS and GTEx e/sQTL studies were calculated as the effect size (β) divided by its standard error. For each GWAS lead variant, an LD window was defined as the chromosomal region containing variants with r^2^ > 0.1 (estimated using the corresponding population from Phase 3 of the 1000 Genomes Project as the reference panel) and extended by an additional 50,000 bp on each side. GWAS–e/sQTL–tissue combinations with a colocalization posterior probability (CLPP) > 0.01 were considered to represent significant colocalization.

### 2.17. fastENLOC Analysis of Genomic Colocalization for Exercise and T2D

To further fine-map genes and regulatory mechanisms (eQTL/sQTL) that may mediate shared risk loci for exercise-related traits and T2D, the Bayesian colocalization method fastENLOC was additionally applied. fastENLOC uses its embedded DAP-G algorithm to perform fine-mapping separately for GWAS and e/sQTL loci, estimating the posterior probability that each variant is causal and then computing the probability that the two signals share the same causal variant (colocalization probability), without imposing an upper bound on the number of independent causal variants per locus.

As input to fastENLOC, Z scores for each variant from the GWAS and GTEx e/sQTL summary statistics were calculated as the effect size (β) divided by its standard error. For each GWAS lead variant, an LD window was defined as the chromosomal region containing variants with r^2^ > 0.1 (estimated using the corresponding population from Phase 3 of the 1000 Genomes Project as the reference panel) and extended by an additional 50,000 bp on each side. Colocalization between exercise and T2D GWAS loci and e/sQTL signals across 49 GTEx tissues was tested, and results were summarized using regional colocalization probabilities (RCPs). Following recommended practice, GWAS–e/sQTL–tissue combinations with RCP > 0.1 were considered to show significant colocalization.

### 2.18. Cell-Type Expression Annotation and Exon-Level Expression Analysis of Prioritized Genes

Cell-type composition across samples was visualized using stacked bar plots and grouped UMAP projections to compare the relative proportions of each cell population between conditions. For prioritized genes identified by fastENLOC, eCAVIAR, open chromatin to gene expression analyses, gsMap, and WGCNA in prioritized cell types, cell-type expression patterns were examined using violin plots and UMAP expression overlays. All results were exported as high-quality image files and corresponding structured data tables.

To further characterize transcript isoform usage, the exon expression module of the GTEx project was used to annotate prioritized genes. This analysis enabled examination of the number and structural features of transcript isoforms and comparison of exon-level expression patterns for these genes across human tissues.

### 2.19. Experimental Animals

Male 10-week-old C57BL/6J mice (Beijing Huafukang Bioscience Co., Ltd., Beijing, China), were housed in a specific pathogen-free facility under controlled conditions (temperature 22 ± 2 °C, relative humidity 50–60%, 12 h light/dark cycle) with ad libitum access to food and water. A T2D model was induced using a high-fat diet contained 60% energy from fat (H10060, Huafukang Bioscience Co., Ltd., Beijing, China). After 1 week of acclimatization, mice were assigned to a normal chow group, a high-fat diet group, and a high-fat diet plus exercise group (n = 6 per group). Group allocation was performed before the intervention using a randomization procedure. The primary experimental outcome was glucose homeostasis, assessed by fasting blood glucose, glucose tolerance test (GTT), and insulin tolerance test (ITT). Secondary outcomes included grip strength, skeletal muscle histology, *Mau2* expression, and *Mau2* intron retention.

No formal a priori sample size calculation was performed. The sample size was determined based on previous comparable mouse exercise studies, experimental feasibility, and the principle of reducing animal use. Therefore, the animal experiments should be interpreted as exploratory rather than fully powered confirmatory experiments.

Mice were included if they completed the dietary and exercise intervention and had available metabolic and tissue samples for downstream analyses. Mice would be excluded before analysis if they died during the intervention, developed severe unrelated illness, or had unusable tissue or RNA samples due to technical failure. No animals were excluded for outcome-based reasons. Histological quantification and image-based analyses were performed by investigators blinded to group allocation.

For terminal tissue collection, mice were anesthetized with inhaled isoflurane. After adequate anesthesia and unconsciousness were confirmed by loss of reflexes, mice were euthanized by cervical dislocation under isoflurane anesthesia before tissue harvest, in order to minimize pain and distress. Injectable anesthetic dosages were not applicable because inhalation anesthesia rather than injectable anesthesia was used in this study.

The exercise protocol consisted of running on treadmill at 10 m/min for 50 min (including warm-up and cool-down), 5 days per week, for 16 weeks. All animal procedures were approved by the Animal Ethics Committee, and were conducted in accordance with institutional guidelines and national regulations for the care and use of laboratory animals.

### 2.20. Glucose and Insulin Tolerance Tests

For the glucose tolerance test (GTT), mice were fasted for 12 h with free access to water and then received an intraperitoneal injection of glucose (2.0 g/kg body weight) dissolved in 0.9% phosphate-buffered saline. For the insulin tolerance test (ITT), mice were fasted for 4 h and then administered an intraperitoneal injection of recombinant human insulin at 1 U/kg body weight. Blood samples were collected from the tail vein at 0 (baseline), 15, 30, 60, 90, and 120 min after injection. Blood glucose levels were measured using a glucometer, and the area under the curve (AUC) of blood glucose over time was calculated to assess whole-body glucose tolerance and insulin sensitivity.

### 2.21. Hematoxylin and Eosin Staining

Gastrocnemius muscle was fixed in 4% paraformaldehyde, dehydrated, embedded in paraffin, and sectioned at 4–5 μm. After deparaffinization and rehydration, sections were stained with hematoxylin and eosin using standard procedures, dehydrated, cleared, and mounted. Images were acquired under a light microscope, and myofiber cross-sectional areas were quantified using Image J (version 2.35; National Institutes of Health, Bethesda, MD, USA).

### 2.22. RNA Sequencing

Total RNA was extracted from gastrocnemius muscle using TRIzol reagent (Invitrogen, Thermo Fisher Scientific, Waltham, MA, USA). RNA concentration and purity were assessed with a NanoDrop 2000 spectrophotometer (Thermo Fisher Scientific, Waltham, MA, USA), and RNA integrity was evaluated using an Agilent 2100 Bioanalyzer (Agilent Technologies, Santa Clara, CA, USA). RNA libraries were prepared with the NEBNext Ultra RNA Library Prep Kit (New England Biolabs, Ipswich, MA, USA). The workflow included poly(A)+ −mRNA enrichment using magnetic beads, fragmentation, first- and second-strand cDNA synthesis, end repair, 3′ end A-tailing, adaptor ligation, and PCR amplification. Library quality was confirmed using an Agilent 2100 Bioanalyzer and a Qubit fluorometer (Thermo Fisher Scientific, Waltham, MA, USA). Qualified libraries were sequenced on an Illumina HiSeq platform in 150 bp paired-end mode, generating approximately 8.28 Gb of sequencing data per sample.

Raw reads were subjected to quality control using SOAPnuke to remove adaptor-contaminated and low-quality reads, yielding clean reads for downstream analysis. Clean reads were aligned to the mouse reference genome Mus musculus GRCm38.p5 using HISAT2, and alignment files were saved in BAM format.

Alternative splicing analysis was performed with rMATS (v4.1.2). BAM files from HISAT2 and the corresponding genome annotation file (GTF format, Mus musculus GRCm38.p5) were used as input. rMATS was run in the mode for experiments with biological replicates, with read length set to 150 bp and the library type specified according to the sequencing protocol. rMATS identifies five major categories of alternative splicing events: skipped exon (SE), retained intron (RI), alternative 5′ splice site (A5SS), alternative 3′ splice site (A3SS), and mutually exclusive exons (MXE). For each event, rMATS estimates the percent spliced in (PSI) for each group and the between-group difference in inclusion levels. Unless otherwise specified, alternative splicing events with a false discovery rate (FDR) < 0.05 and |ΔPSI (ΔIncLevel)| ≥ 0.20 were defined as significantly differential alternative splicing events.

After read alignment, gene-level read counts were quantified using featureCounts, and count matrices were normalized and analyzed for differential gene expression using DESeq2.

### 2.23. RT-qPCR

Total RNA was extracted using a kit (Qiagen, Hilden, Germany) according to the manufacturer’s instructions. A total of 100 ng of RNAs was reverse-transcribed and quantitative RT-PCR was performed by One Step qRT-PCR SYBR Green Kit (Vazyme, Nanjing, China). The comparative cycle-threshold (CT) method was used, and *Actb* served as an endogenous control. The relative levels of target genes were represented as fold of the control group. Primer sequences are listed in Table 1.

### 2.24. Statistical Analysis for Animal Experiments

Animal data are presented as mean ± SEM. Statistical analyses of animal experiments were performed using GraphPad Prism (version 9.5; GraphPad Software, San Diego, CA, USA) and R. Normality was assessed using the Shapiro–Wilk test, and variance homogeneity was assessed using Levene’s test. For comparisons among three groups, one-way ANOVA followed by Tukey’s post hoc test was used when data met the assumptions of normality and homogeneity of variance. When variance homogeneity was not met, Welch’s ANOVA followed by Games–Howell post hoc test was used. For GTT and ITT curves, repeated-measures two-way ANOVA was used with group, time, and group × time interaction as factors. AUC values for GTT and ITT were calculated and compared among groups using one-way ANOVA or Welch’s ANOVA, as appropriate. For RNA-seq-based differential gene expression, DESeq2 was used as described above. Alternative splicing events were analyzed using rMATS, with FDR < 0.05 and |ΔPSI| ≥ 0.20 considered significant. RT-qPCR data were analyzed using the comparative CT method and compared among groups using the same criteria as other endpoint measurements. Multiple comparisons were adjusted using the corresponding post hoc procedures. Exact *p* values were reported where possible, and *p* < 0.05 was considered statistically significant. Given the modest sample size, the animal experiments were interpreted as exploratory biological support rather than fully powered confirmatory validation.

## 3. Results

### 3.1. Genome-Wide Meta-Analysis of Exercise and T2D

By meta-analyzing GWAS summary statistics from independent cohorts for exercise (ebi-a-GCST006100 and ukb-b-8764) and type 2 diabetes (T2D; finngen_R12_T2D and ebi-a-GCST006867), we combined data from a total of 1,952,901 participants to increase statistical power for detecting genetic variants associated with exercise-related traits and T2D. The GWAS for exercise included 810,868 individuals (7,743,637 single nucleotide polymorphisms, SNPs), and the GWAS for T2D included 1,142,033 individuals (9,392,975 SNPs). After obtaining GWAS summary statistics from each cohort, we performed random-effects meta-analyses of effect sizes to mitigate between-cohort heterogeneity and used the resulting combined summary statistics for downstream enrichment and integrative analyses.

### 3.2. Enrichment of Exercise- and T2D-Associated Signals in eQTL and sQTL

**Figure 2:**
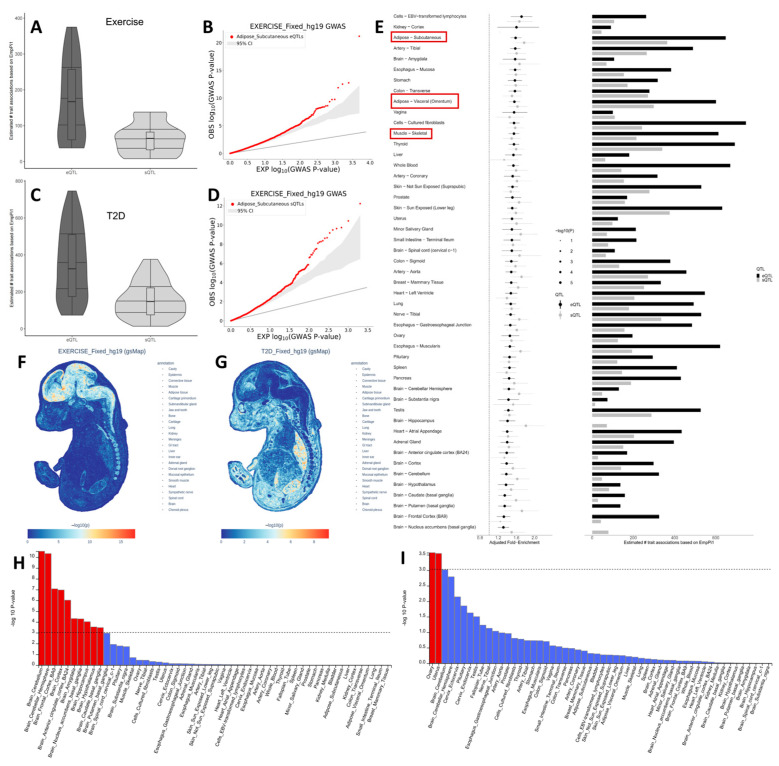
Tissue-level enrichment and spatial localization of GWAS signals for exercise and T2D. (**A**,**C**) Overview of the global distribution of eQTL and sQTL sets used in QTLEnrich analyses for exercise and T2D (violin plots; center line, median; box, interquartile range [IQR]; whiskers, 1.5 × IQR). (**B**,**D**) QTLEnrich quantile–quantile (Q–Q) plots for exercise and T2D using Adipose–Subcutaneous as an example. Red dots indicate observed GWAS −log_10_(*P*) values compared with the expectation under the null; the grey band represents the null interval obtained by resampling. Deviations above the diagonal indicate enrichment. (**E**) Summary of cross-tissue enrichment results. The x-axis shows adjusted fold-enrichment. Bars distinguish eQTL and sQTL. Dot color indicates statistical significance, and dot size reflects the number of QTL variants tested in each tissue. Multiple testing correction was performed according to the QTLEnrich protocol. (**F**,**G**) Organ-level associations after projecting GWAS signals for exercise and T2D onto the E16.5 mouse single-cell spatial transcriptomic atlas. Warmer colors indicate stronger trait–organ association. (**H**,**I**) Enrichment of biological processes at the tissue-compartment level for exercise and T2D (e.g., MAGMA, GeneEnrich, sc-ST analyses). Bar height represents −log_10_(*P*), and the dashed line indicates the false discovery rate (FDR) = 0.05 threshold. Enrichment signals in ovary, uterus, or other reproductive tissues were interpreted cautiously because they may partly reflect known limitations of tissue-reference and QTL-based enrichment analyses rather than direct organ-level causality in T2D. Red boxes indicate the ranked positions of subcutaneous adipose tissue, skeletal muscle, and visceral adipose tissue.

To evaluate the potential organ-level links between eQTL, sQTL, and genetic associations with exercise and T2D, we applied QTLEnrich and accounted for its internal adjustments for enrichment significance and robustness (Figure 2A–D). We systematically tested whether eQTL and sQTL from 49 GTEx v8 tissues were enriched among exercise- and T2D-associated variants (GWAS signals with *p* < 0.05). Across multiple GTEx tissues, including skeletal muscle and adipose depots, both eQTL and sQTL showed significant enrichment for genetic associations with exercise and T2D (Figure 2E). Notably, the strongest enrichment for both sQTL and eQTL was observed in subcutaneous adipose tissue, which contains a high proportion of cell types involved in metabolic regulation and stress responses.

### 3.3. MAGMA Enrichment and Spatial Transcriptomic Mapping Reveal Distinct Tissue Architecture for Exercise and T2D

To investigate the tissue-specific genetic architecture underlying the effects of exercise on diabetes, and to localize exercise and T2D-associated signals at the organ and spatial levels, we first projected both sets of GWAS signals onto the E16.5 mouse embryonic spatial transcriptomic atlas using gsMap (Figure 2F,G). We observed marked spatial enrichment of trait-associated signals in the brain, spinal cord, and sensory nervous system-related structures, whereas enrichment in most non-neural tissues was weak or absent. These patterns suggest that both the central and peripheral nervous systems may act as candidate anatomical contexts associated with exercise-related genetic signals.

We next performed tissue-specific gene-set enrichment analyses using MAGMA (Figure 2H,I). Exercise-related genetic signals showed significant enrichment across multiple brain regions, with the strongest signals in the prefrontal cortex, followed by robust enrichment in the amygdala and hypothalamus; the anterior cingulate cortex and hippocampus were also significantly enriched. In contrast, T2D-associated genetic signals were more prominently concentrated in several abdominal visceral or reproductive tissues, including ovary and uterus. We interpreted these enrichments cautiously, because tissue-enrichment analyses based on GTEx and GWAS-linked regulatory annotations can be influenced by tissue-specific QTL detectability, reproductive-tissue transcriptional complexity, and the broad enrichment tendency of genetic regulatory signals in gonadal tissues; therefore, these signals should not be interpreted as evidence that ovary or uterus are primary pathogenic tissues for T2D.

Overall, spatial localization (gsMap) and tissue-level enrichment (MAGMA) provided mutually reinforcing evidence for the potential relevance of the nervous system to exercise-related genetic architecture, while at the same time revealing divergent tissue targeting between exercise-related traits and T2D.

### 3.4. Gene Function Enrichment Reveals Region-Specific Biological Processes

To dissect the biological functions implicated by genetic signals associated with exercise and T2D, we used GeneEnrich to systematically evaluate functional enrichment of trait-associated genes in specific regions, with a particular focus on subcutaneous adipose tissue and skeletal muscle. Genes located in the major histocompatibility complex (MHC) region were excluded to reduce potential confounding. Using a combination of hypergeometric tests and permutation-based empirical *p* values, we observed significant enrichment of exercise- and T2D-associated genes in multiple gene sets in adipose tissue.

**Figure 3:**
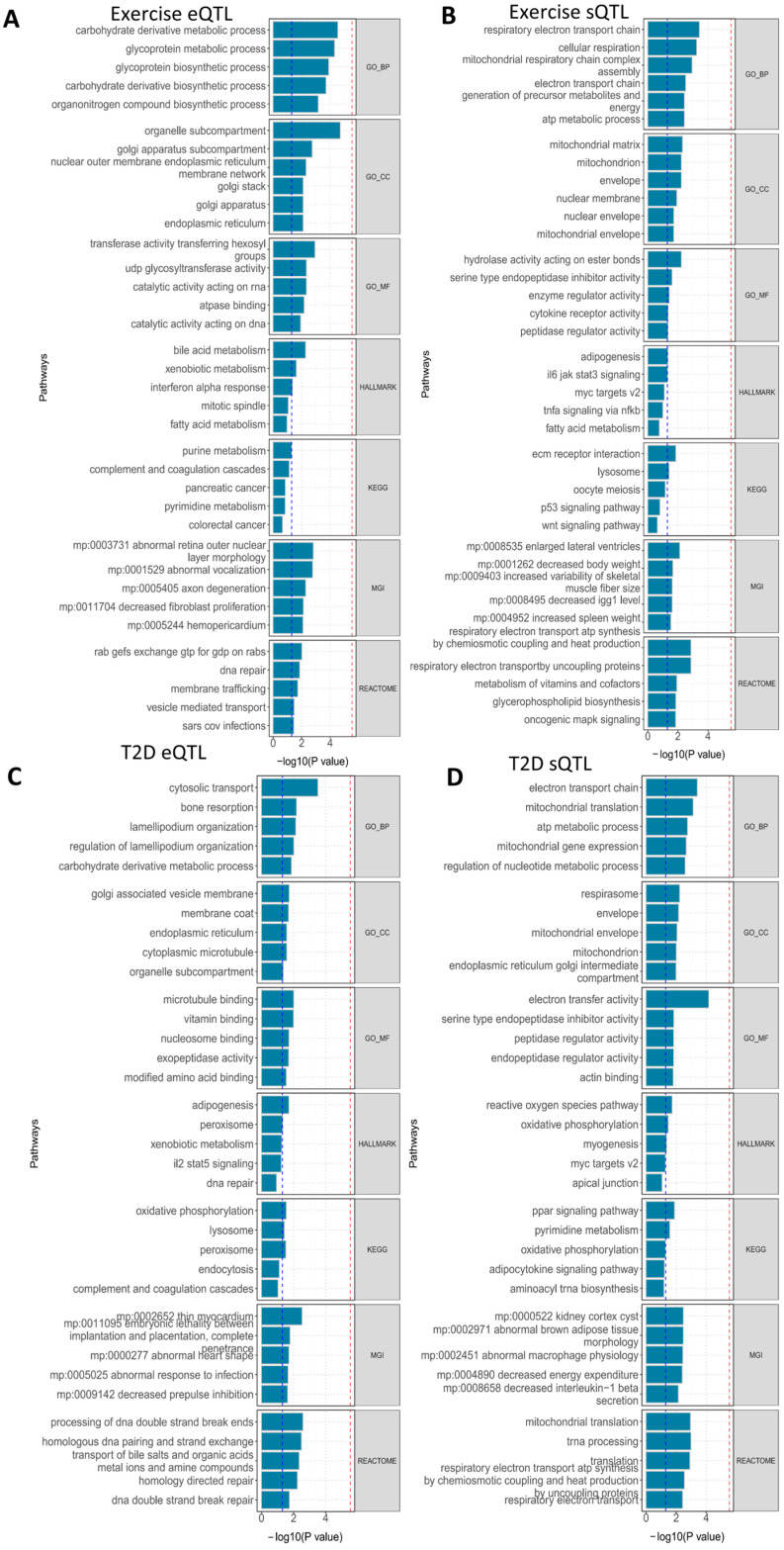
GeneEnrich pathway enrichment for exercise- and T2D-associated eQTL/sQTL genes. (**A**) Exercise eQTL; (**B**) Exercise sQTL; (**C**) T2D eQTL; (**D**) T2D sQTL. Each panel shows the top significantly enriched terms across multiple functional databases, including Gene Ontology biological process (GO_BP), cellular component (GO_CC), and molecular function (GO_MF), HALLMARK, KEGG, Mouse Genome Informatics (MGI), and Reactome. The x-axis represents −log_10_(*P*). The vertical dashed line indicates the significance threshold used in the GeneEnrich analysis.

For exercise, eQTL-associated genes were most strongly enriched in carbohydrate metabolism (Gene Ontology, GO; empirical *p* < 0.05) and glycoprotein metabolism (GO; empirical *p* < 0.05). In contrast, sQTL-associated genes showed particularly striking enrichment in pathways related to the mitochondrial respiratory chain (GO; empirical *p* < 0.05), ATP synthesis and uncoupling protein-mediated thermogenesis (GO; empirical *p* < 0.05), and glycerophospholipid biosynthesis (Reactome; empirical *p* < 0.05). These pathways are closely linked to insulin sensitivity and the browning of white adipose tissue, suggesting that alternative splicing may be one regulatory layer associated with exercise-related adipose tissue remodeling (Figure 3A,B).

In T2D, eQTL-enriched pathways were concentrated in peroxisome and oxidative phosphorylation (KEGG/HALLMARK), endocytosis–lysosome and complement–coagulation cascades (KEGG), and adipogenesis (HALLMARK). sQTL-enriched pathways were even more “mitochondria-centered,” including mitochondrial gene expression and translation, the respiratory chain and respirasome, ATP metabolism and chemiosmotic coupling, and the pathway “respiratory electron transport, ATP synthesis by chemiosmotic coupling, and heat production by uncoupling proteins” (Reactome/GO; empirical *p* < 0.05). We also observed enrichment of PPAR signaling and adipocytokine signaling pathways, reactive oxygen species (ROS)-related pathways, and epithelial junctions, along with Mouse Genome Informatics (MGI) phenotypes linked to energy expenditure, macrophage function, and brown adipose tissue morphology.

Collectively, these findings suggest that the exercise-associated genetic signals in visceral adipose tissue are enriched in pathways related to alternative splicing-dependent regulation of mitochondrial translation, respiratory chain function, thermogenesis, and PPAR/adipokine signaling, which may be linked to energy expenditure, inflammatory regulation, and insulin sensitivity in the context of T2D.

### 3.5. Cellular Composition and Prioritization of Cell Types for Exercise and T2D

Given the central role of the nervous system in energy balance and exercise tolerance, we first used the single-cell eQTL dataset reported by Hao et al. (including eight major cell types such as astrocytes, neurons, and microglia) to construct a cell type-specific Mendelian randomization (csMR) framework. csMR did not identify statistically significant cell type-specific genetic evidence under the current threshold. We therefore extended our analyses to peripheral metabolic tissues by integrating single-cell transcriptomic atlases of skeletal muscle and adipose tissue to systematically evaluate cell types involved in peripheral metabolism.

**Figure 4:**
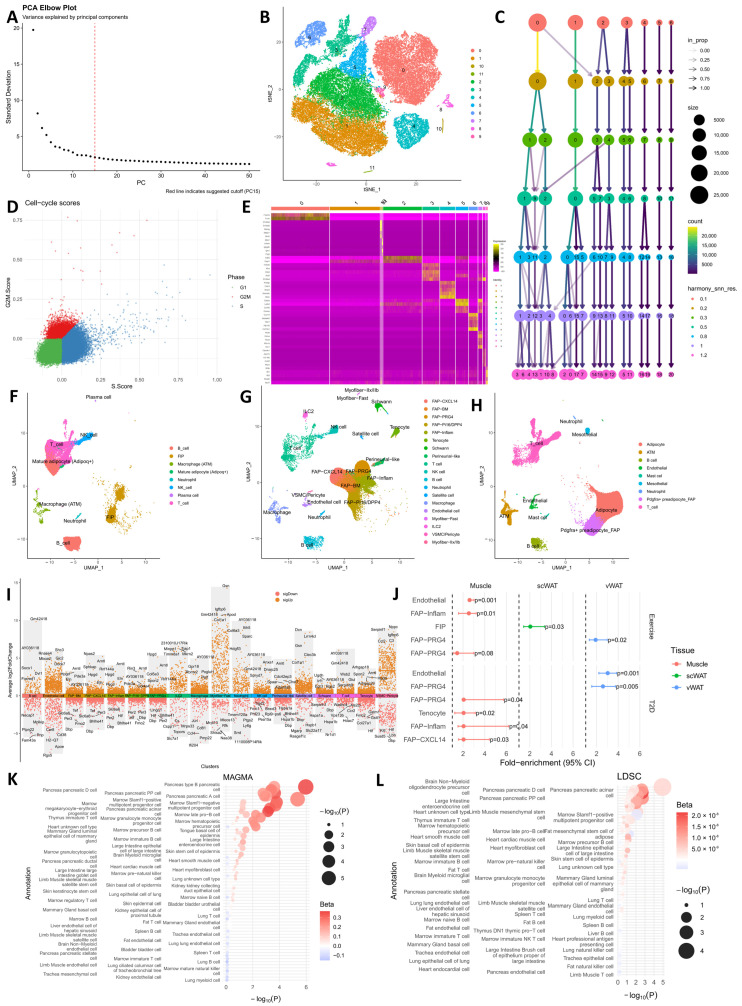
Cellular composition and cell-type analyses for exercise and T2D. (**A**) Principal component analysis (PCA) elbow plot used to determine the number of principal components for downstream analyses (16 PCs selected). (**B**) t-distributed stochastic neighbor embedding (t-SNE) visualization based on Harmony-corrected embeddings, with colors indicating unsupervised clusters. (**C**) Multiresolution clustering tree/dendrogram (node size = number of cells; edges indicate relationships between clusters; side bars depict cluster stability and integration scores across different resolutions). (**D**) Cell-cycle score scatter plot (S phase vs. G2/M phase), illustrating cell-cycle heterogeneity across clusters. (**E**) Heatmap of marker gene expression across clusters (ordered by cluster and gene), used for cell-type annotation and quality assessment. (**F**–**H**) Example UMAP views and annotations of single-cell atlases from three tissues (e.g., (**F**), subcutaneous white adipose tissue, scWAT; (**G**), skeletal muscle; (**H**), visceral white adipose tissue, vWAT), highlighting major cell types such as FAP-Inflam, FAP-PRG4, tenocytes, endothelial cells, and immune cells. (**I**) Schematic Manhattan plot of genome-wide association signals, with prioritized candidate genes/loci indicated for downstream cell type-enrichment analyses. (**J**) Forest plot of cell type-specific enrichment from ECLIPSER, stratified by tissue compartment (muscle, scWAT, vWAT), showing fold-enrichment and 95% confidence intervals for each cell type and for both exercise- and T2D-associated signals. (**K**) Bubble plot summarizing MAGMA gene-set/cell type enrichment results (bubble size = −log_10_(*P*), color = effect direction/β). (**L**) Bubble plot of CELLECT (stratified LD score regression, S-LDSC)-based cell type-specific heritability enrichment. Sample size for the public scRNA-seq dataset was n = 4 mice per group for each tissue.

During the single-cell atlas annotation stage, we performed unsupervised clustering and cell type identification on high-quality cells using Harmony-corrected low-dimensional embeddings. We first assessed dimensionality reduction by principal component analysis (PCA) and elbow plots (Figure 4A), and visualized Harmony-integrated cell distributions using t-distributed stochastic neighbor embedding (t-SNE). The results indicated that batch effects were effectively removed and that cells formed well-defined biological structures in the low-dimensional space (Figure 4B,C). We then quantified cell-cycle states using S phase and G2/M phase scores and observed marked heterogeneity in cell-cycle activity across clusters, with several subpopulations exhibiting pronounced cycling behavior (Figure 4D). Based on the Harmony embeddings, we constructed a cell–cell neighborhood graph and applied a multiresolution clustering strategy (resolution parameters from 0.01 to 3.0, 13 steps in total). By inspecting the cluster tree to evaluate cluster stability across resolutions, we selected a resolution of 0.8 as the optimal parameter for defining subcutaneous adipose cell populations relevant to exercise and T2D. For each cluster, we identified significantly upregulated marker genes, visualized their expression patterns in heatmaps (Figure 4E), and used these markers to annotate cell types and perform differential analyses for downstream investigations (Figure 4F–I).

Using ECLIPSER, we next assessed cell type-specific enrichment of genetic risk signals for exercise and T2D (Figure 4J). Both traits showed significant enrichment in specific cell populations. For exercise, risk loci were predominantly enriched in fibro-adipogenic progenitor (FAP)–Inflam cells in skeletal muscle (fold-enrichment = 2.53, 95% confidence interval [CI]: 1.58–3.21; *p* = 0.001) and in FAP–PRG4 cells (fold-enrichment = 1.45, 95% CI: 1.13–3.00; *p* = 0.023), suggesting that FAP subpopulations may be relevant to exercise-associated genetic signals. In contrast, T2D risk signals were significantly enriched in skeletal muscle tenocytes (fold-enrichment = 2.05, 95% CI: 1.23–3.13; *p* = 0.005), FAP–Inflam cells (fold-enrichment = 2.05, 95% CI: 1.53–6.22; *p* = 0.003), and FAP–PRG4 cells (fold-enrichment = 2.05, 95% CI: 1.92–5.73; *p* = 0.035), as well as in fibro-inflammatory progenitor (FIP) cells in subcutaneous adipose tissue (fold-enrichment = 2.05, 95% CI: 1.43–3.36; *p* = 0.042). Notably, FAP–PRG4 cells showed significant enrichment for both exercise- and T2D-associated signals, suggesting that this subpopulation was might represent one candidate cellular context at the interface between exercise-related signals and T2D-related metabolic biology.

Using the CELLECT framework, analyses (Figure 4K) MAGMA demonstrated that the heritability of exercise-related traits and T2D is specifically enriched in multiple peripheral cell types. Significantly enriched populations included T cells and endothelial cells in adipose tissue, skeletal muscle satellite cells, cardiomyocytes and smooth muscle cells, pancreatic acinar cells, and hematopoietic progenitors in bone marrow (*p* < 0.05 for all). Gene-set analyses with stratified LD score regression (S-LDSC) further validated and extended the S-LDSC results (Figure 4L). T2D risk signals were significantly enriched in several metabolically relevant peripheral cell types, with the strongest associations observed in adipose tissue T cells, endothelial cells, and pancreatic α cells and PP cells (*p* < 0.05). Cardiomyocytes and skeletal muscle satellite cells also showed substantial association signals. Importantly, FAPs also showed nominal enrichment in the MAGMA analyses (*p* < 0.05), which was consistent with the S-LDSC findings and further supported their inclusion as candidate cell populations in downstream analyses.

### 3.6. Integrated Single-Cell Evidence for Cell Type Prioritization

To systematically prioritize candidate cell types associated with exercise- and T2D-related biology, we integrated four complementary lines of computational evidence: (1) single-cell atlas-based annotation, used to identify transcriptionally active cell populations in skeletal muscle and adipose tissue; (2) cell type-specific colocalization and Mendelian randomization (csMR), used to test for genetic evidence supporting cell type-specific involvement; (3) ECLIPSER, which evaluates enrichment of GWAS signals in cell type-specific regulatory elements based on functional genomic annotations; and (4) CELLECT, which combines stratified LD score regression (S-LDSC) and MAGMA-based analyses to assess the contribution of cell type-specific gene expression to trait heritability. Because these methods differ in statistical assumptions and output metrics, we used an evidence-counting approach rather than a formal weighted meta-analysis. The scoring was based on predefined evidence units: the single-cell atlas contributed up to 2 points because skeletal muscle and adipose tissue were counted as separate tissue-level evidence units, whereas csMR, ECLIPSER, and CELLECT each contributed up to 1 point according to their prespecified support criteria. Thus, the total score reflected the number of positive evidence units supporting each candidate cell type, rather than a formal statistical weight or causal estimate.

Using this evidence-counting scheme, fibro–adipogenic progenitor (FAP) cells and endothelial cells received the highest prioritization scores among the evaluated cell populations (Table 2). Their higher scores reflected support from both skeletal muscle and adipose single-cell atlases, together with additional support from ECLIPSER and CELLECT. These results suggest that FAPs and endothelial cells represent candidate cell types supported by cross-tissue and cross-method convergence in exercise- and T2D-related biology.

### 3.7. Candidate Gene Network

**Figure 5:**
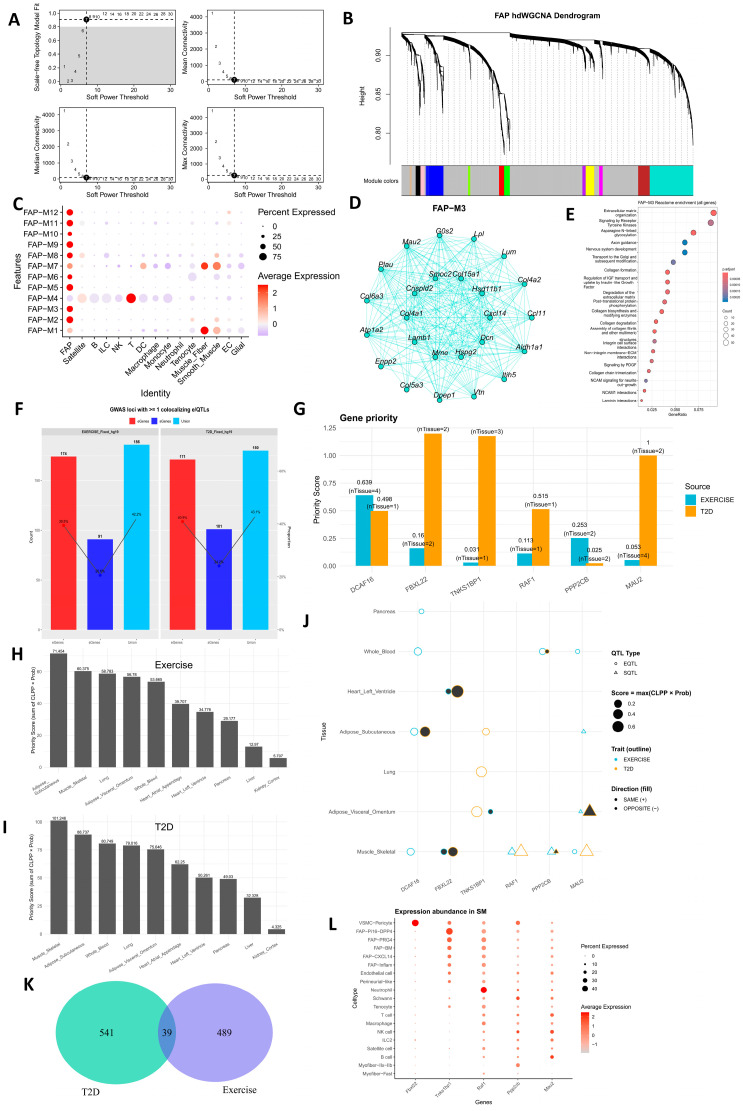
Co-expression network, candidate gene prioritization, and cross-tissue colocalization. (**A**) Soft-threshold selection for hdWGCNA: scale-free topology and mean connectivity were evaluated on metacells (k = 25), and a soft-thresholding power of β = 4 was chosen. (**B**) Gene-level hierarchical clustering tree of FAP–PRG4 cells and dynamically cut co-expression modules (color bar). (**C**) Bubble plot of module eigengene-associated marker gene expression across major cell types (circle area, percentage of expressing cells; color, average expression level). (**D**) Visualization of the co-expression network and hub genes within the M3 module. (**E**) Reactome pathway enrichment analysis for genes in the M3 module. (**F**) Number and proportion of GWAS loci with ≥ 1 colocalizing eQTL/sQTL for exercise and T2D (see Methods for details). (**G**–**I**) Distribution of priority scores summarized by tissue for exercise and T2D, respectively (see Methods for the definition of the priority score). (**H**) Venn diagram showing the overlap of top-priority colocalized genes between exercise and T2D (intersection n = 39). (**J**) Bubble plot of cross-tissue colocalization across tissue–gene pairs. Point shape indicates QTL type (eQTL or sQTL). The outline color denotes the trait (exercise or T2D). Fill color indicates whether GWAS and QTL effects are concordant or discordant in direction. Point size reflects colocalization strength, defined as the maximum value of (CLPP + RCP) across all tested colocalization signals for each tissue–gene pair. (**K**) Venn diagram of shared colocalized genes between exercise and T2D. (**L**) Expression abundance of 5 candidate genes in different cell types.

Fibro–adipogenic progenitor (FAP) cells were prioritized as a candidate cell population potentially involved in both exercise-related traits and T2D. Fibro–adipogenic progenitor (FAP) cells were prioritized as a candidate cell population potentially involved in both exercise-related traits and T2D. To further characterize the transcriptional regulatory programs of FAPs, we performed weighted gene co-expression network analysis using the hdWGCNA framework. To mitigate the intrinsic sparsity of single-cell data, we aggregated cells into metacells (k = 25). We selected a soft-thresholding power of 4 (Figure 5A) to approximate a scale-free topology and to construct a signed topological overlap matrix (TOM). Using a dynamic tree-cutting algorithm, we built a co-expression network and identified 12 distinct co-expression modules (Figure 5B). The hub genes within these modules showed strong FAP specificity (Figure 5C). Among them, the M3 module was predominantly enriched for extracellular matrix (ECM)- and collagen-related pathways, including extracellular matrix organization; collagen formation, degradation, biosynthesis, and modification; integrin- and non-integrin-mediated cell–ECM interactions; and receptor tyrosine kinase, platelet-derived growth factor (PDGF), and insulin-like growth factor (IGF) signaling pathways.

Using eCAVIAR, we identified genes and regulatory variants showing colocalization between GWAS signals and expression QTLs. In total, we detected significant colocalization between GWAS signals and expression QTLs for 1069 genes (CLPP > 0.01). After ranking loci by the product of CLPP and posterior probability (CLPP × Prob), we prioritized 39 overlapping candidate genes shared between exercise and T2D (Figure 5E–L), suggesting partially overlapping regulatory loci between the two traits. Among these, *MAU2* showed a high priority score (Priority score = 1, *n*Tissue = 2; Figure 5G) and exhibited concordant colocalization patterns in visceral adipose tissue and skeletal muscle (Figure 5J). These results nominated *MAU2* as a candidate gene linked to exercise- and T2D-associated regulatory signals. Notably, the *MAU2* signal was largely driven by sQTL, suggesting that alternative splicing may be relevant to its prioritization.

### 3.8. Cell-Type Expression Annotation and Exon-Level Splicing Analysis of Prioritized Genes

**Figure 6:**
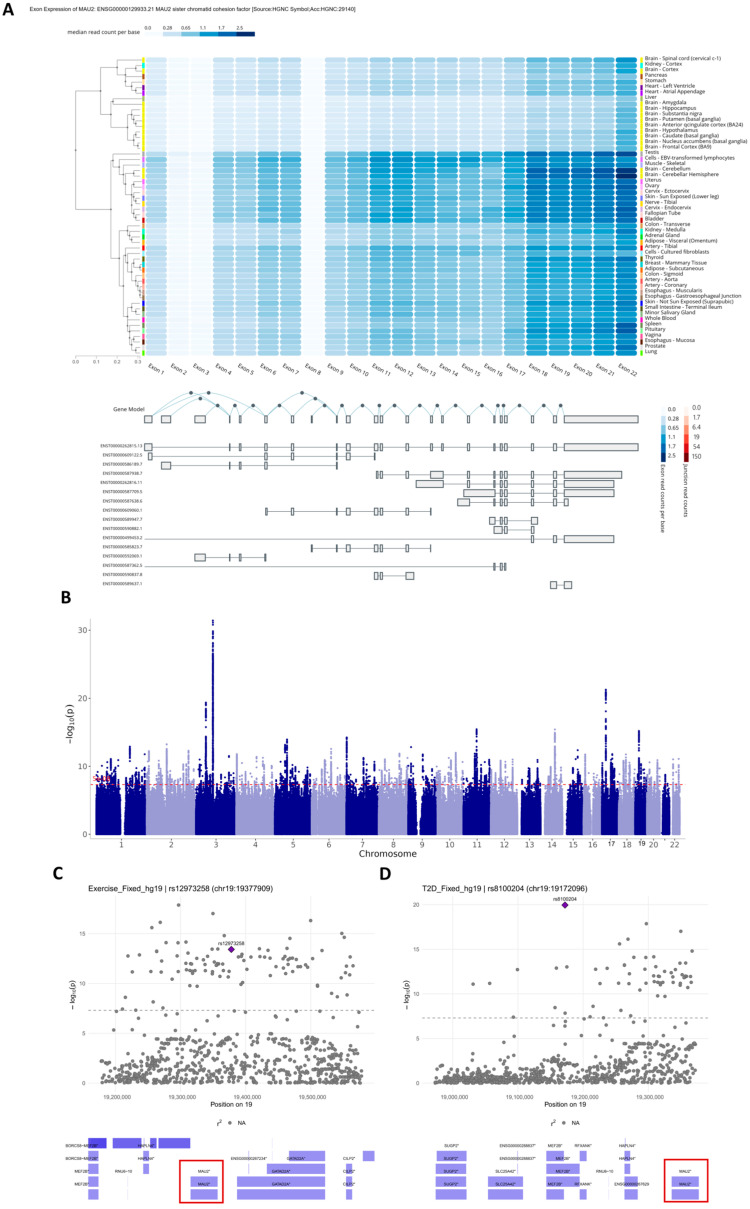
Exon-level expression profile of *Mau2* and regional association signals. (**A**) Exon-level heatmap of *Mau2* across multiple tissues from the GTEx project. Each column represents one exon, and color intensity indicates the median read count per base. The left panel shows hierarchical clustering of tissues, the right panel lists tissue names, and the bottom panel depicts Ensembl transcript models for *Mau2*. (**B**) Genome-wide Manhattan plot of the T2D GWAS (hg19). The red dashed line indicates the genome-wide significance threshold (P = 5 × 10−8). (**C**,**D**) Regional association and colocalization plots for the *Mau2* lead SNPs in the exercise- and T2D-related GWAS, respectively. The red box highlights the position of *Mau2*.

To further annotate the cellular expression patterns of prioritized candidate genes, we integrated multiple lines of prioritization evidence—including eCAVIAR colocalization, gsMap spatial mapping, and hub genes identified by hdWGCNA—and selected *Mau2* as a representative gene for in-depth annotation. *Mau2* was expressed across multiple cell types but showed higher expression levels in immune cell populations (Figure 5L). At the exon level, *MAU2* exhibited relatively high overall expression in skeletal muscle, heart, and arterial tissues, with lower expression in most other organs (Figure 6A). Notably, its elevated expression in skeletal muscle coincided with a tissue-specific alternative splicing event involving exons 10–15 (Figure 6B). This observation suggests that tissue-specific splicing regulation of *Mau2* splicing may represent a candidate molecular feature associated with exercise- and T2D-related regulatory programs.

We next identified 860 independent, trait-associated sentinel SNPs that were distributed across the genome, with a notable enrichment on chromosome 3. Using conditional and joint analysis (COJO) implemented in GCTA, we further resolved two independent secondary signals at selected loci (e.g., rs19377909 and rs19172096), which emerged as lead SNPs for in both the exercise- and T2D-related analyses (Figure 6C,D). These findings indicate that multiple functional variants may reside within the same locus, highlighting the complexity of the genetic architecture underlying these traits.

### 3.9. Exercise Reduces Mau2 Intron Retention in Skeletal Muscle of Diabetic Mice

**Figure 7:**
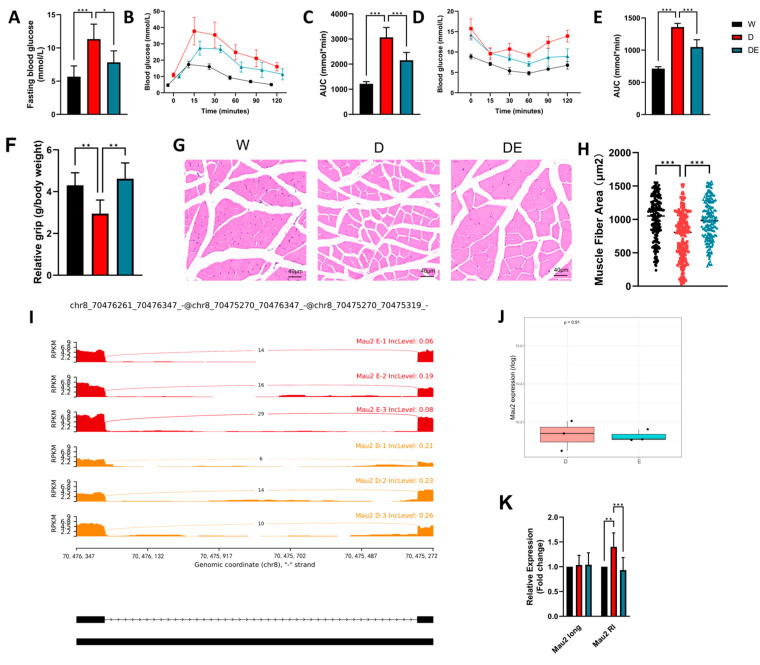
Exercise reduces *Mau2* intron retention in gastrocnemius muscle of diabetic mice. (**A**–**H**) Phenotypic and histological analyses were performed with n = 6 mice per group. (**I**–**K**) RNA-seq and RT-qPCR analyses were performed with n = 3 mice per group. (**A**) Fasting blood glucose. (**B**,**C**) Glucose tolerance test curves and corresponding area under the curve. (**D**,**E**) Insulin tolerance test curves and AUC. (**F**) Relative grip strength. (**G**,**H**) H&E staining of gastrocnemius muscle and muscle fiber area. Scale bar = 40 μm. (**I**) Alternative splicing analysis of *Mau2* showing altered intron retention in response to exercise. (**J**) RNA-seq rlog-normalized expression levels of *Mau2* in gastrocnemius muscle. (**K**) RT–qPCR analysis of the two *Mau2* transcript isoforms. Data are presented as mean ± SEM. GTT and ITT curves were analyzed using repeated-measures two-way ANOVA. AUC values and endpoint measurements were analyzed using one-way ANOVA followed by post hoc multiple-comparison tests, as described in the Methods. * *p* < 0.05, ** *p* < 0.01, *** *p* < 0.001.

To experimentally examine selected findings from the integrative analyses, we subjected diabetic mice to a 16-week aerobic exercise intervention. Aerobic exercise significantly reduced fasting blood glucose in diabetic mice (D vs. DE: mean difference = 3.50, 95% CI: 0.67 to 6.33, Tukey-adjusted *p* = 0.02) and improved glucose tolerance, as reflected by a reduced GTT AUC compared with diabetic mice (D vs. DE: mean difference = 913.8, 95% CI: 472.2 to 1355, Tukey-adjusted *p* < 0.001; Figure 7A–C). Consistently, the ITT AUC also differed significantly among groups (ordinary one-way ANOVA, F(2, 15) = 108.0, *p* < 0.001, η^2^ = 0.935), supporting improved insulin sensitivity after exercise (Figure 7D, E). In gastrocnemius muscle, exercise alleviated diabetes-associated muscle atrophy, as indicated by increased myofiber cross-sectional area compared with diabetic mice (one-way ANOVA, F(2, 551) = 35.80, *p* < 0.001, η^2^ = 0.115; D vs. DE: mean difference = −238.2, 95% CI: −318.4 to −158.0, Tukey-adjusted *p* < 0.001). The DE group was no longer significantly different from the W group in myofiber cross-sectional area (W vs. DE: Tukey-adjusted *p* = 0.77). Exercise also increased relative grip strength in diabetic mice (one-way ANOVA, F (2,15) = 10.43, *p* = 0.001, η^2^ = 0.582; Figure 7F–H).

We next examined alternative splicing events in gastrocnemius muscle by RNA-seq. Exercise reduced intron retention of *Mau2* in diabetic mice (Figure 7I), whereas the overall expression level of *Mau2* was not significantly altered (Figure 7J). This exercise-associated reduction in *Mau2* intron retention was further supported by RT–qPCR, with a significant isoform-by-group interaction detected by two-way ANOVA (F(2, 29) = 4.576, *p* = 0.02; Figure 7K). These findings indicate that *Mau2* intron retention is an exercise-responsive splicing event associated with improved muscle morphology, although they do not by themselves establish a causal role for *Mau2* in muscle atrophy.

## 4. Discussion

Type 2 diabetes (T2D) is a systemic metabolic disease characterized by insulin resistance and impaired β-cell function, with long-term cardiovascular and cerebrovascular complications making it one of the major global public health burdens. The etiology of T2D is highly complex, arising from intricate interactions between strong genetic predisposition and substantial environmental risk factors [23,24,25]. Large-scale genomic studies have identified hundreds of loci associated with T2D risk [26,27]. However, translating these statistical associations into biologically interpretable tissues, cell types, and regulatory programs remains a major challenge. The present study aimed to address this gap by establishing a multiscale integrative framework linking population-level genetic signals with tissue-, cell-type-, and gene-level regulatory features. By integrating large-scale GWAS meta-analyses, multi-tissue regulatory QTL maps, single-cell-resolution atlases of skeletal muscle and adipose tissue, and spatial transcriptomic data, we systematically examined how exercise- and T2D-associated signals converge across multiple biological layers. These analyses identified non-random enrichment patterns across tissues, cell types, molecular pathways, and spatial contexts: (1) the genetic risks of exercise-related traits and T2D show non-random convergence in key metabolic organs, underscoring the dominant role of peripheral metabolic tissues in mediating the benefits of exercise; (2) fibro–adipogenic progenitors (FAPs) emerge as candidate cell populations potentially linked to exercise- and T2D-related tissue remodeling; (3) *MAU2/Mau2* and its alternative splicing emerged as candidate molecular features associated with exercise-related metabolic remodeling and T2D-related regulatory programs.

### 4.1. Convergence of Genetic Risk in Metabolic Tissues Suggests Peripheral Regulatory Involvement

Our study first suggests, at the organ level, that the genetic risks of exercise-related traits and T2D display marked tissue and spatial specificity. This specificity is unlikely to be incidental; rather, it points directly to distinct tissue origins underlying different behavioral and metabolic phenotypes. Previous PA GWAS have consistently identified CADM2 as one of the strongest loci associated with physical activity, with additional links to obesity and metabolic traits. In the present study, we focused on regulatory convergence between exercise-related genetic signals and T2D across tissue, cell-type, QTL, colocalization, and experimental layers [19]. Therefore, our prioritization of FAP-related programs and *Mau2*/Mau2 splicing does not contradict the established importance of CADM2; rather, it suggests that different components of PA genetics may act at different biological levels.

We found that genetic risk signals for both exercise and T2D are most prominently enriched in skeletal muscle and adipose tissue. Skeletal muscle is the primary site of insulin-dependent glucose uptake and oxidation, and its mitochondrial function, capacity for glucose–lipid metabolism, and fiber-type composition collectively determine whole-body glucose clearance and exercise tolerance. Systemic impairment of these functions is a hallmark of T2D and exercise intolerance [28,29,30]. Our results suggest that the shared genetic architecture of exercise and T2D is, to a large extent, rooted in the metabolic vulnerability of skeletal muscle. Specifically, risk variants are likely to act by disrupting key pathways active in this tissue, including the mitochondrial respiratory chain, fatty acid β-oxidation, and insulin signaling [31,32]. Such organ-level metabolic dysfunction directly blunts skeletal muscle responses to both exercise and insulin, thereby limiting its ability to clear circulating glucose and lipids and ultimately manifesting as insulin resistance, impaired glucose tolerance, and reduced exercise capacity—core clinical features of T2D. In parallel, T2D genetic risk also shows selective convergence in visceral adipose tissue and other visceral metabolic organs. Visceral fat is a central hub for regulating fatty acid release, inflammatory cytokine secretion, and multiple endocrine signals, and its functional integrity critically shapes the metabolic load experienced by the liver and skeletal muscle [33,34,35]. Our analyses indicate that pathways related to lipid mobilization, lipid droplet turnover, and inflammatory responses are particularly enriched for T2D-associated variants in visceral adipose tissue. When such variants perturb the expression or splicing of these genes, they are expected to enhance systemic free fatty acid spillover and chronic low-grade inflammation, promote ectopic lipid deposition, and amplify insulin resistance in the liver and skeletal muscle, thereby driving hyperglycemia, dyslipidemia, and cardiovascular complications characteristic of T2D.

Taken together, the organ-specific links in genetic risk between exercise and T2D—especially at the level of splicing regulation—suggest a possible coordinated imbalance between skeletal muscle, the main “glucose-clearing organ,” and visceral adipose tissue, an important “metabolic pressure source.” Diminished metabolic responsiveness in skeletal muscle, coupled with exaggerated lipid and inflammatory output from visceral fat, may contribute to both the clinical severity of T2D and the heterogeneity in patients’ responses to exercise-based interventions.

### 4.2. FAP-Associated Extracellular Matrix Programs in Exercise–T2D Tissue Remodeling

Fibro–adipogenic progenitors (FAPs) are known to participate in extracellular matrix (ECM) organization and tissue remodeling in skeletal muscle and adipose tissue [36,37]. This capacity may contribute to the structural integrity and plasticity of these tissues. Our hdWGCNA analysis showed that important co-expression modules within FAPs are significantly enriched for pathways related to collagen synthesis and degradation, ECM–receptor interaction, and IGF/PDGF signaling. This pattern suggests that FAP-associated programs may be involved in matrix organization, cell adhesion, and local growth-factor signaling. Integrating these findings with our eQTL/sQTL enrichment and colocalization results, we suggest that T2D-associated risk variants, together with a high-fat diet environment, might be linked to FAP-associated ECM programs with pro-fibrotic or pro-stiffness features. These changes could be associated with collagen deposition, interstitial remodeling, and structural alterations at the fat–muscle interface. In turn, such changes might affect glucose and insulin diffusion within the tissue, muscle contractile efficiency, and fatty acid oxidation, thereby contributing to insulin resistance, ectopic lipid accumulation, and reduced exercise tolerance. Conversely, exercise-related signals, together with exercise-induced mechanical loading and metabolic stress, might be associated with FAP programs related to matrix turnover and cytoskeletal remodeling. These programs could contribute to ECM renewal and tissue plasticity in skeletal muscle and adipose tissue.

### 4.3. Mau2/Mau2 Splicing as a Candidate Molecular Feature Linking Exercise- and T2D-Related Regulatory Signals

At the gene and network level, we used eCAVIAR, fastENLOC, and hdWGCNA to link population-level genetic signals to cell-intrinsic co-expression modules, and from hundreds of colocalized genes we prioritized *MAU2/Mau2* as a candidate gene. Unlike classical “metabolic genes,” *Mau2* is best known for its role in sister chromatid cohesion and maintenance of higher-order chromosome structure, yet it has also been implicated as a susceptibility locus for metabolic syndrome [38,39]. In our data, its genetic associations were predominantly driven by sQTL and showed concordant patterns of colocalization with exercise- and T2D-related signals in both skeletal muscle and adipose tissue.

Within FAP-enriched co-expression modules, *Mau2* was associated with pathways related to ECM and cell–matrix adhesion, as well as RNA processing and chromatin organization. This combination of features suggests that *MAU2/Mau2* may be linked to chromatin organization and splicing-related programs, thereby potentially contributing to coordinated regulation of metabolic and structural genes. Along the exercise–T2D axis, *MAU2/Mau2* might therefore represent a candidate molecular feature connecting structural, transcriptional, and splicing-related regulation.

The observed change in *Mau2* intron retention may be biologically relevant because intron retention can alter transcript processing, nuclear export, nonsense-mediated decay, or isoform usage, thereby changing gene function without necessarily changing total gene expression. In our mouse data, exercise reduced *Mau2* intron retention while total Mau2 expression remained largely unchanged, suggesting a splicing-level regulatory effect rather than a simple expression-level change [40]. Given the association of *Mau2* with chromatin organization and RNA-processing modules, this splicing event may be linked to broader transcriptional and structural remodeling in diabetic skeletal muscle. However, we interpret this as a candidate mechanism rather than direct proof that *Mau2* intron retention causes muscle atrophy.

This concept is consistent with the broader notion of exercise-induced epigenetic remodeling: epigenetic and chromatin-level changes tend to confer relatively durable effects at the tissue level, whereas the transcription of metabolic genes is more susceptible to transient environmental and stress-related stimuli.

### 4.4. Limitations

The animal experiments were exploratory because no formal a priori sample size calculation was performed and the sample size was modest, particularly for RNA-seq and splicing analyses. In addition, validation was limited to an HFD-induced diabetic mouse model, and whether similar Mau2 splicing changes occur in ob/ob or db/db mice remains unknown. Finally, although *MAU2/Mau2* splicing was supported by colocalization, tissue-level splicing signals, RNA-seq, and RT-qPCR, we did not perform knockdown, overexpression, or splice-isoform-specific rescue experiments. Thus, Mau2 intron retention should be interpreted as a candidate splicing event rather than a confirmed causal mechanism. Future studies using larger cohorts and cell type- or gene-specific perturbation models are needed to clarify its functional role in diabetic muscle remodeling.

## 5. Conclusions

In summary, by integrating large-scale GWAS, cross-tissue eQTL/sQTL data, spatial and single-cell transcriptomics, and mouse exercise experiments, we established a multiscale framework for examining exercise-related regulatory programs in T2D. We identified skeletal muscle and adipose tissue as prioritized tissues and highlighted FAP-associated microenvironmental remodeling and *MAU2/Mau2* splicing as candidate cellular and molecular features. These findings generate testable hypotheses for future studies using larger cohorts and gene- or cell type-specific perturbation models.

## Figures and Tables

**Table 1:** Primers used for RT-qPCR.

Gene Name	Forward Primer	Reverse Primer
*Mau2* long	CATGTGGGAACGCCATGGAT	GGGGGTGGGCCATCGTAA
*Mau2* RI	CACCTGACCTCATTGCACC	CTCCATCAACAGCAGCTAGA
*Actb*	CCAACCGTGAAAAGATGACC	ACCAGAGGCATACAGGGACA

**Table 2:** Integrated single-cell evidence-based prioritization of cell types for exercise- and T2D-related trait.

Cell Type	Single-Cell Atlas Skeleton Muscle	Single-Cell Atlas Adipose Tissue	csMR	ECLIPSER	CELLECT	Total Score
FAP	1	1	0	1	1	4
Endothelial cells	1	1	0	1	1	4
T cells	0	0	0	1	1	2
FIP	1	0	0	0	1	2
Pancreas-related cells	0	0	0	1	1	2

Note: The total score represents an evidence-counting prioritization index rather than a formal statistical weight. The single-cell atlas evidence was counted by tissue, with skeletal muscle and adipose tissue treated as two predefined tissue-level evidence units; therefore, this layer could contribute 0, 1, or 2 points. csMR, ECLIPSER, and CELLECT each contributed 1 point when the prespecified support criterion was met and 0 points otherwise. Across all evidence units, a score of 0 indicated absence of positive support under the predefined criterion and was not treated as negative evidence or as a penalty. The total score therefore reflects the number of positive evidence units supporting each cell type and was used only for candidate prioritization, not as a causal probability, effect-size estimate, or meta-analytic result.

## Data Availability

All publicly available datasets used in this study are listed in Supplementary Table S1. The key analysis scripts and parameter settings are available at: https://github.com/xiaojingzhe1120/CODE_for_cells-15-00979 (accessed on 20 May 2026).

## Supplementary Materials

The following supporting information can be downloaded at: https://www.mdpi.com/article/10.3390/cells15110979/s1. Table S1. Data Resource.

## Abbreviations

The following abbreviations are used in this manuscript:

T2D	Type 2 diabetes
GWAS	Genome-wide association study
SNP	Single-nucleotide polymorphism
eQTL	Expression quantitative trait locus
sQTL	Splicing quantitative trait locus
GTEx	Genotype-Tissue Expression
scRNA-seq	Single-cell RNA sequencing
sc-ST	Single-cell spatial transcriptomics
csMR	Cell type-specific Mendelian randomization
FAPs	Fibro-adipogenic progenitors
QTLEnrich	Quantitative trait locus enrichment analysis
MAGMA	Multi-marker Analysis of GenoMic Annotation
ECLIPSER	Enrichment of Causal Loci and Identification of Relevant Cell Types in Single Cell Expression and Regulation data
CELLECT	Cell type Expression-specific integration for Complex Traits
hdWGCNA	High-dimensional weighted gene co-expression network analysis
eCAVIAR	Exact Causal Variants Identification in Associated Regions
FUMA	Functional Mapping and Annotation
GCTA-COJO	Genome-wide Complex Trait Analysis—Conditional and Joint analysis
CLPP	Colocalization posterior probability
RCP	Regional colocalization probability
MAF	Minor allele frequency
LD	Linkage disequilibrium
MHC	Major histocompatibility complex
GO	Gene Ontology
KEGG	Kyoto Encyclopedia of Genes and Genomes
MSigDB	Molecular Signatures Database
MGI	Mouse Genome Informatics
PCA	Principal component analysis
t-SNE	t-distributed stochastic neighbor embedding
UMI	Unique molecular identifier
GEO	Gene Expression Omnibus
RNA-seq	RNA sequencing
RT-qPCR	Reverse transcription quantitative polymerase chain reaction
GTT	Glucose tolerance test
ITT	Insulin tolerance test
AUC	Area under the curve
H&E	Hematoxylin and eosin
FDR	False discovery rate
PSI	Percent spliced in
SE	Skipped exon
RI	Retained intron
A5SS	Alternative 5′ splice site
A3SS	Alternative 3′ splice site
MXE	Mutually exclusive exons
ECM	Extracellular matrix
PDGF	Platelet-derived growth factor
IGF	Insulin-like growth factor
